# Spinal Lesions as Clinical Manifestations of Plasma Cell Neoplasia

**DOI:** 10.3390/curroncol29090490

**Published:** 2022-08-29

**Authors:** Lea Baumgart, Melanie Barz, Claire Delbridge, Amir Kaywan Aftahy, Insa Katrin Janssen, Philipp J. Jost, Yu-Mi Ryang, Bernhard Meyer, Jens Gempt

**Affiliations:** 1 Department of Neurosurgery, Klinikum rechts der Isar, School of Medicine, Technical University Munich, 81675 Munich, Germany; 2 Department of Neuropathology, Klinikum rechts der Isar, School of Medicine, Technical University Munich, 81675 Munich, Germany; 3 Department of Neurosurgery, Hôpitaux Universitaires de Genève, 1205 Genève, Switzerland; 4 III. Medical Department of Hematology and Oncology, Klinikum rechts der Isar, School of Medicine, Tech-nical University Munich, 81675 Munich, Germany; 5 Division of Oncology, Department of Internal Medicine, Medical University of Graz, 8036 Graz, Austria; 6 Department of Neurosurgery, Helios Klinikum Berlin Buch, 13125 Berlin, Germany

**Keywords:** solitary bone plasmacytoma, multiple myeloma, survival, spine, surgery

## Abstract

(1) Background: Plasma cell neoplasia can be separated into independent subtypes including multiple myeloma (MM) and solitary plasmacytoma of the bone (SBP). The first clinical signs patients present with are skeletal pain, most commonly involving ribs and vertebrae. (2) Methods: Retrospective analysis of 114 patients (38 female, 76 male) receiving spinal surgery from March 2006 until April 2020. Neurological impairments and surgical instability were the criteria for intervention in this cohort. Analysis was based on demographic data, Spinal Instability Neoplastic Score (SINS), location of the lesion, spinal levels of tumor involvement, surgical treatment, histopathological workup, adjuvant therapy, functional outcome, and overall survival (OS). (3) Results: The following surgical procedures were performed: posterior stabilization only in 9 patients, posterior stabilization and decompression without vertebral body replacement in 56 patients, tumor debulking and decompression only in 8 patients, anterior approach in combined approach without vertebral body replacement and without biopsy and/or without kyphoplasty in 33 patients, 3 patients received biopsies only, and 5 patients received kyphoplasty only. The histopathology diagnoses were MM in 94 cases and SBP in 20 cases. Median OS was 72 months (53.4–90.6 months). Preoperative KPSS was 80% (range 40–100%), the postoperative KPSS was 80% (range 50–100%). (4) Conclusions: Surgery for patients with plasma cell neoplasia is beneficial in case of neurological impairment and spinal instability. Moreover, we were able to show that patients with MM and a low number of spinal levels to be supplied have a better prognosis as well as a younger age at the time of the surgical intervention.

## 1. Introduction

Plasma cell neoplasia is the second most common hematological malignancy following non-Hodgkin lymphoma [1,2,3]. It accounts for 1% of all cancers and approximately 10% of all hematologic malignancies [3]. 

The two subtypes of plasma cell neoplasia are solitary bone plasmacytoma (SBP) and extramedullary plasmacytoma. SBP has a 3-year probability of progression to multiple myeloma (MM) by 10.1% [3,4]. 

MM is slightly more common in men than in women, and is twice as common in African-Americans compared with Caucasians [5]. In general, it predominantly impacts older adults with a median age at diagnosis of 69 years [6,7,8]. Survival estimates in MM vary based on eligibility for ASCT. If so, the 4-year survival rate is more than 80% and the median OS is approximately 8 years [9]. Among elderly patients (age > 75 years), median OS is approximately 5 years [10]. A more precise estimation of prognosis requires an assessment of multiple factors [11,12], starting with Durie–Salmon Staging [13] and the international Staging System (ISS) [14,15,16] to reflect the tumor burden in MM followed by the molecular subtype of MM. In particular, the presence or absence of secondary cytogenetic abnormalities such as del (17p), gain (1q), or del (1p) [17,18] should be assessed.

Additionally, there are two other factors associated with aggressive disease: elevated serum lactate dehydrogenase and evidence of circulating plasma cells on routine peripheral smear examination. To combine both into tumor burden and disease biology, Palumbo et al. published a revised ISS in 2015 [16].

The first clinical signs that patients present with are osteolytic bone lesions, fractures, bone pain, progressive anemia, hypercalcemia, renal insufficiency, recurrent infections, and/or bleeding [19,20,21]. In case of osteolytic bone lesions, vertebral involvement is prevalent in 60–80% of MM patients [22]. The SIN Score is used to detect affected vertebral bodies which may become weakened with progressive bone destruction or defects in the posterior wall and pedicles, which means that a fracture may compromise spinal stability and lead to neurological impairment. It is divided into six categories starting with localization, load-dependent pain, and bone lesion and ending with radiological spine formation, vertebral body collapse and post-lateral involvement. All categories are scored from 0–2 or 3 depending on severity. Depending on the score obtained, the lesion is rated as stable (SINS score 1–6), potentially unstable (SINS score 7–12), and unstable (SINS score 13–18) [23].

In recent studies, the necessity of surgery in the case of spinal involvement due to plasma cell neoplasia has been discussed. They all reached the same conclusion: in case of structural instability and neurological impairment, surgery must be considered [23,24,25]. To date, little is known about the surgical treatment and long-term prognosis of patients with plasma cell neoplasia. Thus, we aimed in this study to investigate the surgical outcome of these patients as well as to find factors which may impact long-term survival.

## 2. Materials and Methods

### 2.1. Patients and Methods

**Figure 1:**
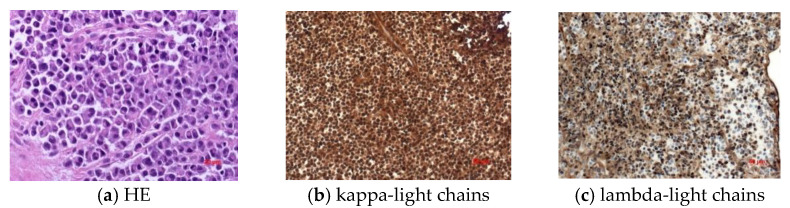
Immunohistochemical work-up to show kappa light chains (**b**) as well as lambda light chains (**c**) compared to standard HE staining (**a**).

A total of 114 patients with spinal lesions as the first clinical manifestation of plasma cell neoplasia and characterized by spinal instability or neurological impairment treated between March 2006 and April 2020 were reviewed retrospectively. A histopathological workup confirmed 94 patients with a diagnosis of multiple myeloma (MM) and 20 patients with solitary plasmacytoma of the bone (SPB) (Figure 1). The basic characteristics of patients are listed in Table 1. 

Neurological impairments and/or surgical instability were the criteria for intervention in this cohort. Analysis was performed based on age, gender, location of the lesion, spinal levels of tumor involvement, surgical treatment, histopathological workup, functional outcome, and overall survival. Patients’ quality of life was assessed by using pre- and postoperative the Karnofsky Performance Status Scale (KPSS). OS was calculated from the initial diagnosis of plasma cell neoplasia until death or censored at the time of last follow-up. Furthermore, we calculated the overall survival from the time of surgery. The neurologic status of the patient was assessed pre- and postoperatively, according to the American Spinal Injury Association (ASIA) impairment scale. The ASIA impairment scale was rated on a scale from grade A–E, representing the patient’s motor and sensory function with grade A representing no motor and sensory function in the sacral segments S4–5 and grade E representing a normal motor and sensory function in all spinal segments. 

### 2.2. Statistics

Statistical analysis was performed using STATA Version 13.1 (2011, StataCorp, College Station, TX, USA). In the descriptive data analysis, we show non-normally distributed data as median and interquartile range (IR) and normally distributed variables as mean and standard deviation. Univariate OS distributions were compared using Kaplan–Meier estimates (Log-rank). Multivariate survival analysis was performed using a Cox proportional hazard regression model. In the univariate analysis, we included sex, age, KPSS at admission and day of discharge, SIN-Score, adjuvant radio- and chemotherapy, duration of hospitalization, ASA-Score, SIN-Score, and number of spinal levels supplied. In multivariate Cox regression analysis, the following parameters were included: sex, KPSS at admission, SIN-Score, and number of spinal levels supplied. Differences with a type one error probability of less than 0.05 were considered statistically significant.

### 2.3. Ethics Approval

The study was approved by the local ethics committee (N°335-16S) of the Technical University Munich, School of Medicine. It was conducted in accordance with the ethical standards of the 1964 Declaration of Helsinki and its later amendments [26].

## 3. Results

This retrospective study analyzed 114 cases of spinal plasma cell neoplasia surgically managed in our tertiary care institute over a 14-year period (2006–2020) (Table 1). Among these patients, 76 (66.67%) patients were males and 38 (33.33%) were females with a median age of 65.44 years (range 35.65–87.92 years). The median ASA was 2 (range 1–4). Median Karnofsky Performance Status Score (KPSS) at surgery was 80 % (range 40–100%). 

At hospitalization, physical examinations showed neurological deficits or palsies resulting from spinal cord compression for 45 patients (39.48%) and pain in 97 patients (85.09%). The median OS was 72 months (53.4–90.6 months). A total of 36 patients died during follow-up. Median OS from the time of surgery was 58 months (32.2–83.7 months).Among all patients, 52 had one spine lesion, while 24 patients presented two lesions and 38 presented three or more spinal lesions.

### 3.1. Overall Survival

#### 3.1.1. Univariate Analysis

Subgroup analysis of patients older than 60 years independent of their diagnosis shows significantly longer hospitalization (*p* = 0.000; CI: 95% 9.301319–482.6197) and shorter survival time (*p* = 0.001; CI: 95% 0.2664898–0.7264819).

#### 3.1.2. Multivariate Analysis

The following parameters were included in the Cox regression analysis: sex, KPSS at admission, SIN-Score, and number of spinal levels supplied. For patients with multiple myeloma, only the number of spinal levels supplied were significantly related to OS (*p* = 0.000, HR: 1.149641; CI: 95% 1.06378–1.242431). In the case of SPB as diagnosis, no significance was shown.

**Figure 2:**
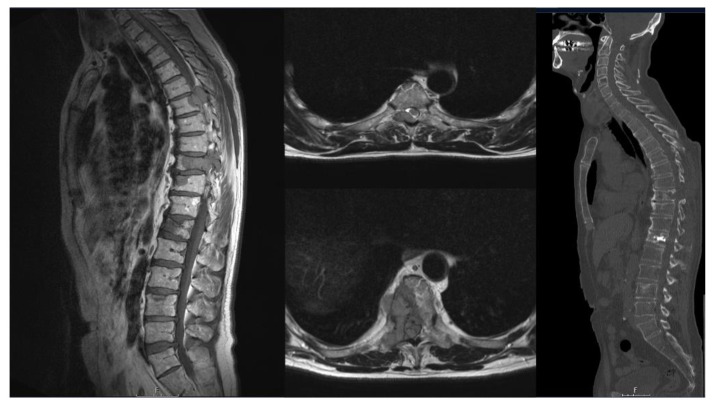
A 60-year-old patient presented with radical pain corresponding to dermatomes Th6 and Th9, which had been present for weeks.

The main manifestation of tumor lesions was thoracic spine in 35 patients, followed by the thoraco-lumbar junction in 23 patients (Table 2). To identify possible spinal instability due to lesions of plasma cell neoplasia, imaging was performed as the first way to calculate the SIN-Score as previously described [27]. Analogous to the SIN-Score, we have divided our patients into 3 groups: spinal stability (0–6), indeterminate stability (7–12), and instability (13–18) (Table 1). A total of 9 patients received posterior stabilization only, posterior stabilization and decompression without vertebral body replacement was performed in 56 patients, tumor debulking and decompression only in 8 patients (Figure 2), anterior approach in combined approach without vertebral body replacement and without biopsy and/or without kyphoplasty in 33 patients, 3 patients received biopsy only, and 5 patients received a kyphoplasty only. 

After the surgical treatment, the discussion of each individual case took place within the framework of a neuro-oncology board. Together with the colleagues from radiotherapy and the medical oncologist, the decision on further therapy was made as follows: a total of 27 patients received postoperative adjuvant radiotherapy alone, while 29 patients received chemotherapy alone, and 30 received both chemotherapy and radiotherapy (Table 3).

If postoperative adjuvant therapy is divided according to histology, 18 patients with MM received radiotherapy alone, 27 patients received chemotherapy alone, and 27 patients received a combined adjuvant therapy. In case of SBP, nine patients received radiotherapy alone, two patients received chemotherapy alone and three patients received a combined adjuvant therapy (Table 3). Patients who underwent postoperative radiotherapy had significantly longer median OS (*p* = 0.029): 86 (range 58.6–113.4) vs. 62 (range 10.7–113.3) months. No significance was found for postoperative chemotherapy. In multivariate Cox regression analysis, postoperative radiotherapy was proven to be a significant protective factor for longer OS (HR= −1.068, *p* = 0.011).

T1 SE-weighted sagittal (A) and T1 FS-weighted axial (B, C) magnetic resonance images showed tumor suspected lesions at Th6 and Th9. Additionally, MRI showed pathological fractures at Th12 and L3. Postoperative CT-imaging (D) confirmed decompression via hemilaminectomy of Th6- and right Th9-vertebral body as well as vertebroplasty of Th9 and Th12. 

Histology confirmed the rare case of multiple myeloma with plasma cell leukemia.

## 4. Discussion

Multiple myeloma and SBP are common malignant primary tumors in vertebra and usually treated conservatively with chemotherapy and radiotherapy [23,28]. However, at the time of diagnosis, 1–2% of patients suffer from anemia, hypercalcemia, renal failure, or infections before spinal involvement [29]. As conservative treatment of multiple myeloma advances and improved imaging makes skeletal lesions easier to detect, patients may progress to an advanced disease state, with spinal lesions that cause neurological deficits becoming more prominent [30]. The treatment of multiple myeloma and SBPs in the case of an initial spinal manifestation is still not clearly defined unless there is spinal instability or a neurological deficit at that time. Although there is still no golden standard for the treatment of this special group of patients, surgery is inevitable [23,31,32,33]. 

The analysis of this cohort represents the data of 20 SPB and 94 multiple myelomas. Of these, 77 patients had an initial manifestation of the disease in the spine with neurological deficits, unstable fractures, and pain. In 23 cases, the operation was urgent, and in 6 cases it was an emergency. However, apart from the need to provide surgical care for this cohort, out of these 77 initial diagnoses, 57 patients showed a systemic indication for treatment due to further manifestations in the entire skeleton and/or due to the results from the bone marrow biopsy. 

In the case of an emergency indication due to neurological deficits, laminectomy initially became established. 

Via this access, one can remove posterior elements of the spinal column, but less of a tumor, and this often fails to achieve immediate decompression [34]. 

However, in order to produce less blood loss and smaller wounds, tumor debulking and decompression as already described by Qian et al. has proven effective. In our study, this approach was used in eight patients. According to this, faster access to adjuvant therapy is possible. However, this approach is only possible in the absence of spinal instability and can therefore not be considered a standard of care [35]. 

Over the years, the operational possibilities have developed significantly [36]. 

In combination with the imaging developments and the SIN-Score established by them, the approach via decompression of the spinal tumor and stabilization has been shown to be a great gain in treatment in the case of spinal instability, pain, and/or neurological deficit [37]. However, as we know, implants can become infected, loosen, and cause recurrence of symptoms, especially in patients whose immune systems have been compromised [30]. Alternatively, vertebroplasty or kyphoplasty can be performed if the spine is stable. This can be used to treat the pain symptoms and to take a biopsy in order to obtain a histology in the case of initial manifestation of a spinal lesion [30]. In this study population, kyphoplasty was indicated in five cases. These patients showed an improvement in pain symptoms. However, this could not be shown in combination with dorsal stabilization in our cohort.

In our study, postoperative radiotherapy proved to be a significant factor for longer median OS. This high sensitivity to radiotherapy, has also been described in recent studies [38,39,40,41]. Therefore, it justifiably has its raison d’être in adjuvant therapy of MM and SBP.

Apart from surgical options, systemic therapies have improved survival. In the 1990s, a high-dose of melphalan followed by an autologous stem cell transplant (HDM-ASCT) was the standard of care for patients aged <65 years [9,42,43], there was a shift to thalidomide in 2001–2008 [44]. Due to the side effects of this drug and the development of lenalidomide, it was discontinued in 2008 [45]. In parallel, bortezomib was developed in 2005 and received approval as a treatment for this disease [46]. VAD (vincristin, adriamycin, and dexamethason), another DNA-damaging agent, was used in regimes [47]. In our cohort, 20 patients received VAD after surgery.

Another significant step was taken in 2016 and 2017 when second-generation proteasome inhibitors such as carfilzomib and ixazomib received approval as well as daratumumab and panobinostat. Other drugs such as pomalidomide, daratumumab, and panobinostat also received approval in this time frame [7]. The adjuvant treatment in this cohort was also carried out according to these guidelines. However, lenalidomide was largely initiated as second-line therapy at our center. 

In summary, a multimodal concept should be implemented including surgical treatment and subsequent systemic therapy. 

### Study Limitations

This study offers a single-center experience only. A limitation of this study is its retrospective nature, as it introduces an unavoidable selection bias. Furthermore, due to the highly selected patient population, the number of patients included in this study is relatively small. Moreover, this study cannot reflect the impact of cytogenetic or molecular biological status on the prognosis. Therefore, most recent innovations in systemic chemotherapy are not reflected. 

## 5. Conclusions

Surgery for patients with plasma cell neoplasia is beneficial in the case of neurological impairment and spinal instability. Moreover, we were able to show that patients with MM and a low number of spinal levels to be supplied have a better prognosis as well as a younger age at the time of their operations.

## Figures and Tables

**Table 1:** Demographic and clinical data overview.

Demographics *n* (%) or Mean/Median	Multiple Myeloma	Solitary Bone Plasmocytoma	Total
Age	64.29 years	64.18 years	64.27 years
Sex	62 m/32 f	14 m/6 f	76 m/38 f
Clinical presentation preoperative			
KPSS	80%	90%	80%
ASIA A	0	0	0
ASIA B	6 (6.38%)	2 (10.00%)	8 (7.02%)
ASIA C	9 (9.57%)	2 (10.00%)	11 (9.65%)
ASIA D	20 (21.28%)	6 (30.00%)	26 (22.81%)
ASIA E	59 (62.77%)	10 (50.00%)	69 (60.53%)
SINS, *n* (%)			
Median	8	8	8
Mean	8	8	8
Stable	20 (21.27%)	4 (20.00%)	24 (21.05%)
Indeterminate	68 (72.35%)	15 (75.00%)	83 (72.81%)
Instable	6 (6.38%)	1 (5.00%)	7 (6.14%)
Clinical presentation postoperative			
KPSS	80%	90%	80%
ASIA A	0	0	0
ASIA B	1 (1.06%)	0	1 (0.88%)
ASIA C	9 (9.57%)	4 (20.00%)	13 (11.40%)
ASIA D	22 (23.40%)	6 (30.00%)	28 (24.56%)
ASIA E	62 (65.96%)	10 (50.00%)	72 (63.16%)

**Table 2:** Localization of tumor lesions.

Location *n* (%) or Mean/Median	Multiple Myeloma	Solitary Bone Plasmocytoma	Total
Cervical	12 (12.77%)	5 (25.00%)	17 (14.91%)
Thoracic	26 (27.66%)	9 (45.00%)	35 (30.70%)
Lumbar	11 (11.70%)	1 (5.00%)	12 (10.53%)
Sacral	2 (2.13%)	1 (5.00%)	3 (2.63%)
Cervico-thoracic	17 (18.09%)	3 (15.00%)	20 (17.54%)
Thoraco-lumbar	22 (23.40%)	1 (5.00%)	23 (20.18%)
Lumbo-sacral	1 (1.06%)	0	1 (0.88%)
Thoraco-lumbo-sacral	2 (2.13%)	0	2 (1.75%)
Cervical and lumbar	1 (1.06%)	0	1 (0.88%)

**Table 3:** Adjuvant therapy divided according to multiple myeloma and solitary bone plasmacytoma.

Adjuvant Treatment * *n* (%) or Mean/Median	Multiple Myeloma	Solitary Bone Plasmocytoma	Total
Chemoimmune therapy alone	27 (28.72%)	2 (10.00%)	29 (25.44%)
Radiotherapy alone	18 (19.15%)	9 (45.00%)	27 (23.68%)
Chemoimmune therapy+ radiotherapy	27 (28.72%)	3 (15.00%)	30 (26.32%)
Unknown	21 (22.34%)	6 (30.00%)	27 (23.68%)
Antibody therapy	1 (1.06%)	0	1 (0.88%)

* Data based solely on neuro-oncology board recommendations.

## Data Availability

Data and material are not publicly available. The data for this manuscript can be obtained from the author upon reasonable request.
